# Found Down for How Long?

**DOI:** 10.51894/001c.165606

**Published:** 2026-07-31

**Authors:** Roman Gernat, Garrett Tomaski, James P. McCarty

**Affiliations:** 1 Henry Ford - Jackson

## 84

### Introduction

Compartment syndrome is a serious and difficult to diagnose pathology infrequently seen in the emergency department. The consequences of missed compartment syndrome include limb threatening complications to local vasculature, musculature, and innervation - potentially life threatening infections or renal failure can develop as well.

### Case Description

A mid thirties female PMHx of substance abuse who presents to the ER by EMS for altered mental status. Per EMS, the patient was supposed to check out of a hotel room today and they were called because she had not checked out. The patient was found in a room surrounded by bottles of alcohol as well as a rolled up dollar bill. On arrival, the patient complained of bilateral leg pain and back pain as well as a possible head injury. The patient’s clinical course was complicated by a CPK as high as 355,000 which ultimately led to fasciotomies of bilateral lower extremities and left lower extremity as well as an amputation of the LLE prior to patient ultimately leaving AMA after a prolonged hospital course.

### Discussion

When evaluating a patient for altered mental status in the ER some or all of the story should be suspect. In any patient with an unclear down time, rhabdomyolysis should be on your list of differentials. In a patient that is found down the pathophysiology for rhabdo is an occlusion of blood flow in an affected limb that causes subsequent muscular ischemia and damage. Cases of severe rhabdomyolysis should have you considering the possibility of compartment syndrome in a patient so it is important to do a thorough physical exam in these patients to evaluate for soft compartments.

